# Feasibility, Safety and Reliability of Surgeon-Directed Transcranial Motor Evoked Potentials Monitoring in Scoliosis Surgery

**DOI:** 10.3390/children10091560

**Published:** 2023-09-15

**Authors:** Aude Kerdoncuff, Patrice Henry, Roxane Compagnon, Franck Accadbled, Jérôme Sales de Gauzy, Tristan Langlais

**Affiliations:** 1 Department of Paediatric Orthopedic Surgery, Children’s Hospital, Toulouse University, 31062 Toulouse, France; kerdoncuffa@outlook.fr (A.K.); roxane.compagnon@gmail.com (R.C.); accadbled.f@chu-toulouse.fr (F.A.); salesdegauzy.j@chu-toulouse.fr (J.S.d.G.); 2 Department of Neurology, Purpan Hospital, Toulouse University, 31062 Toulouse, France; henry.p@chu-toulouse.fr

**Keywords:** scoliosis, multimodal spinal cord intra operative monitoring, pediatric spinal surgery, complex spine deformities

## Abstract

(1) Background: Neuromonitoring is essential in corrective surgery for scoliosis. Our aim was to assess the feasibility, safety and reliability of “surgeon-directed” intraoperative monitoring transcranial motor evoked potentials (MEP) of patients. (2) Methods: A retrospective single-center study of a cohort of 190 scoliosis surgeries, monitored by NIM ECLIPSE (Medtronic), between 2017 and 2021. Girls (144) and boys (46) (mean age of 15 years) were included. There were 149 idiopathic and 41 secondary scoliosis. The monitoring consisted of stimulating the primary motor cortex to record the MEP with muscular recording on the thenar, vastus lateralis, tibialis anterior and adductor hallucis muscles. (3) Results: The monitoring data was usable in 180 cases (94.7%), with 178 true negatives, no false negatives and one false positive. There was one true positive case. The predictive negative value was 100%. The monitoring data was unusable in 10 cases (i.e., three idiopathic and seven secondary scoliosis). (4) Conclusions: Simplified transcranial MEP monitoring known as “surgeon-directed module” is usable, safety and reliable in surgery for moderate scoliosis. It is feasible in 95% of cases with a negative predictive value of 100%.

## 1. Introduction

Scoliosis surgery carries a risk of neurological complications estimated at between 0.35% and 1% of cases [1,2,3]. This complication rate can be as high as 9% in certain congenital deformities [4]. Intraoperative neuromonitoring is necessary to reduce this risk [5] and has been recommended by the SRS since 1992 [6]. Several monitoring methods have been proposed, but the gold standard is multimodal monitoring, which analyzes somesthetic evoked potentials (SEPs) and motor evoked potentials (MEPs) performed by a doctor specializing in electrophysiology [7]. SEPs were described in the 1970s and assess the ascending sensory pathways whereas MEPs were described in the 1980s and directly analyze the descending motor pathways [8]. MEP procedure uses electrical or magnetic stimulation of the cortex or spinal cord to produce signals in the corticospinal pathways [8]. Some teams [9,10] combine the two techniques (MEPs and SEPs) to achieve reliable neuromonitoring. However, most teams do not have a permanent electrophysiologist due to a lack of resources and funding. To compensate for the lack of electrophysiologists, simplified monitoring techniques (i.e., MEPs only)—known as surgeon-directed modules—have recently been developed that are easy to use and interpret [11,12]. They allow the surgical team to perform neurological monitoring in the absence of an electrophysiologist. The aim of our study was to assess the feasibility, safety and reliability of this surgeon-directed module technique, based on a cohort of scoliosis operations in children, adolescents and young adults.

## 2. Materials and Methods

### 2.1. Design and Population Criteria

**Figure 1:**
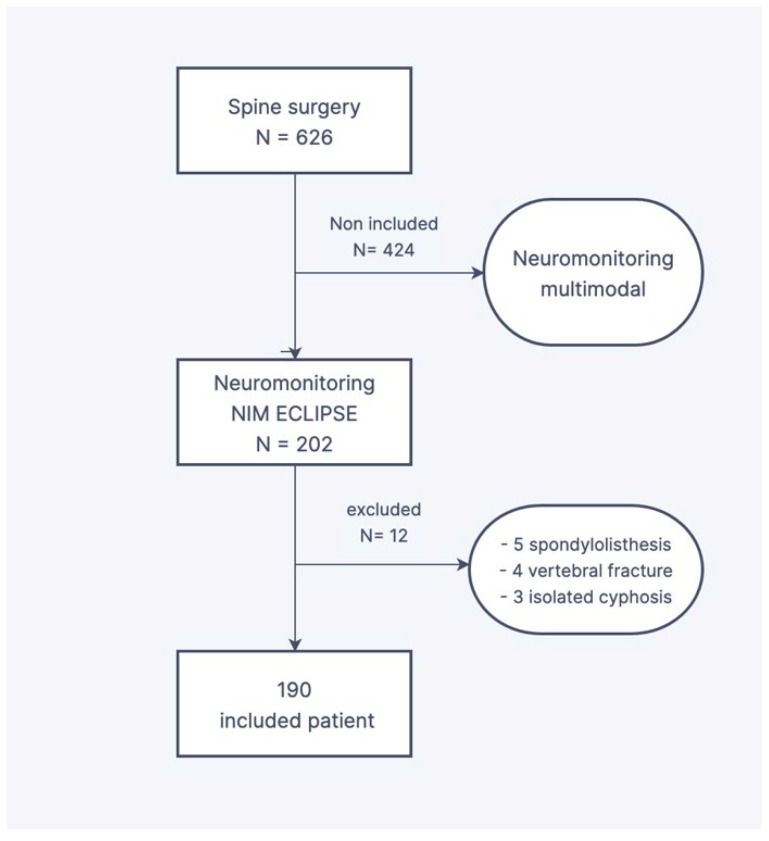
Flow chart.

This is a single-center retrospective study based on the records of operated spinal deformities from 2017 to 2021. The inclusion criteria were idiopathic or secondary scoliosis who underwent a posterior correction-fusion or posterior growing rods and a neuromonitoring performed by the surgeon only. The exclusion criteria were scoliosis surgery with monitoring by an electrophysiologist, corrections of isolated kyphosis or spondylolisthesis, and vertebral fracture treatment. The patient’s records monitoring was made possible by the computerized patient file, completed progressively by the referring surgeon. Of the 626 spinal surgical procedures conducted over the inclusion period, the monitoring was surgeon-directed in 190 consecutive cases (Figure 1).

The mean age was 15 years and 3 months (SD = 3 years, range from 5 to 26 years). The cohort comprised 144 girls and 46 boys. One hundred and forty-nine idiopathic scoliosis and 41 secondary scoliosis were included. This secondary scoliosis included: 14 cerebral palsy, two spinal muscular atrophies, six myopathies, nine congenital deformities (two of which were operated on as part of a poly-malformities syndrome with associated cardiopathy), one Marfan syndrome, one hypophosphatemia rickets, two Prader-Willi syndromes, two arthrogryposis, one Scheuermann disease, two syndromic obesities with hypopituitarism and one neurofibromatosis. The radiographic characteristic of the deformity is reported in Table 1. For the patients included in our cohort, the number of times monitoring had been possible was observed and the number of true and false negatives and true and false positives reported. The tracings were analyzed by the surgeon during the operation and by an electrophysiologist at a distance. Surgeons were trained by the neurophysiologist in the correct positioning of the electrodes, how to monitor them and how to be vigilant for anomalies during the procedure. Monitoring failures at the start of the system’s implementation are linked to the learning curve for monitoring. In addition, all patients were operated on by two surgeons specializing in the management of spinal deformities in children (JSDG and FA) and with considerable technique expertise. The surgical technique for spinal arthrodesis depended on the type of curvature. A hybrid construct using at proximal a supra-transverse hook and pedicle hook clamp (or pedicle screw) [13], sublaminar band in the thoracic area [14], and distal lumbar screws in cases of thoracic curvature. In cases of lumbar or thoracotomy-lumbar curvature, an “all-screw” construct was used.

### 2.2. Neuromonitoring Procedure

**Figure 2:**
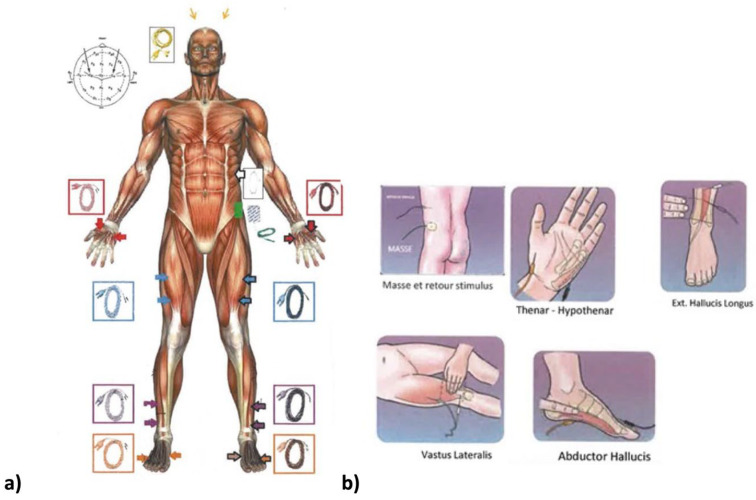
Electrode schemes. (**a**) Electrode sites and color code. Color and white go to right, color and black go to left. Red are thenar sites; blue are vastus lateralis; purple is tibialis anterior; and orange are abductor hallucis. Yellow electrodes go to right and left motor cortex area. The green electrode, called “earth ground”, is placed on the iliac crest. (**b**) Focus on each electrode site.

**Figure 3:**
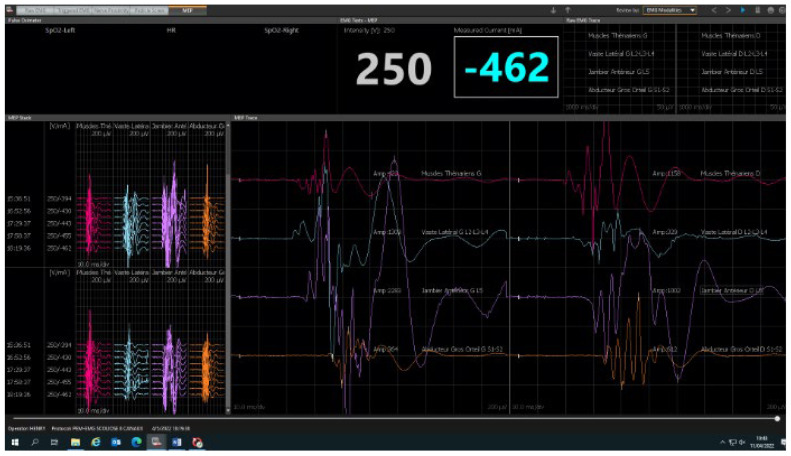
Normal neuromonitoring. Red curves corresponded to the thenar muscular responses; blue curves to the vastus lateralis muscular responses; purple curves to the tibialis anterior muscular responses; and the orange curves to the abductor hallucis muscular responses.

**Figure 4:**
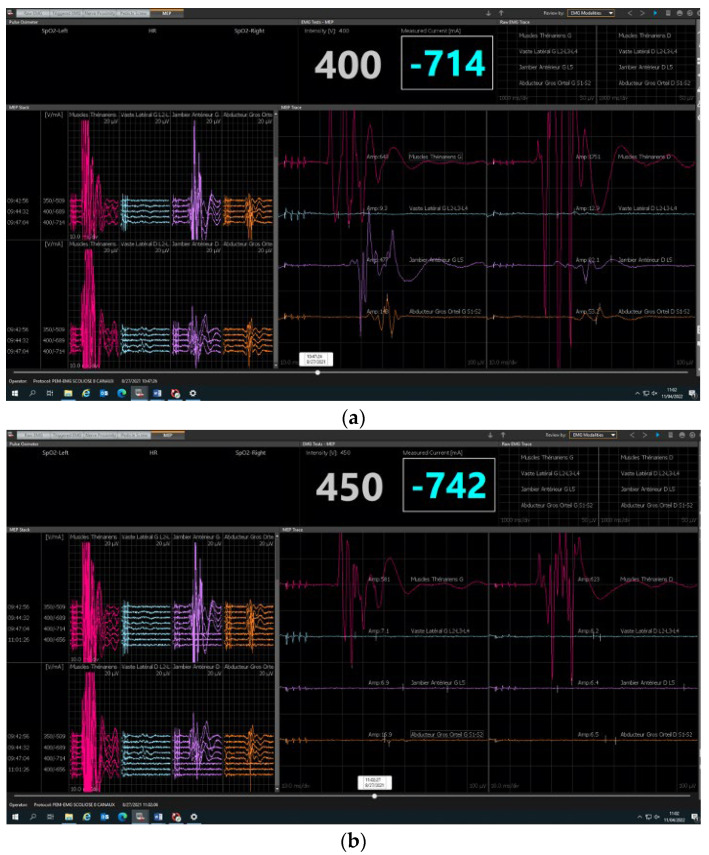
(**a**) Baseline before incision (**b**) True positive: Neuromonitoring alert, loss of all responses except upper limb.

Neuromonitoring was conducted using the integrated “surgeon-directed” module on the NIM ECLIPSE device (Medtronic, Minneapolis, MN, USA). Only the integrity of the pyramidal motor pathway is explored by the transcortical electrical stimulation of the primary motor cortex with recording of the motor responses at the distal muscular effectors in the hands and lower limbs. The control panel on the NIM ECLIPSE device displays any faulty connection or other fault. The stimulation parameters (train, number of impulses in the train, interval between each electric shock, etc.) and the reception parameters (bandwidths) are preset by the manufacturer. Only the stimulation intensity and the recorded MEP response signal amplitude can be changed by the surgeon user. The stimulation intensity is gradually increased until satisfactory responses are obtained. It ranges from 150 to 450 volts and rarely exceeds 500 volts. Safety features measure the amperage delivered to the scalp. In our center, we use between 250 and 350 volts on average. The maximum amplitude obtained is specific to each patient and determines the choice of the useful stimulation intensity. There is a great variability between individuals in MEP amplitudes from 0.050 to 500 millivolts and for some muscles and patients’ maximum amplitudes of 1 to 4 volts. It is important to adapt the stimulations in young children and patients with seizure, especially if this is poorly controlled, with stimulations that stay low by precaution. After anesthetic induction, the electrodes are positioned by the surgeon before the patient is turned prone (Figure 2). The recording electrodes (muscles) are positioned on the lower limbs (adductor hallucis, tibialis anterior, vastus lateralis muscles) and on the upper limbs (thenar eminence). The stimulation electrodes are positioned on the projection regions on the scalp of the primary motor cortex. As for all electrophysiological procedures, a neutral electrode, known as the “earth ground”, must be positioned on one of the iliac crests. The electrodes placed on the upper limbs serve as controls to detect false positives. A first stimulation is conducted before incision with patient under analgesia to obtain a baseline and verify that the monitoring system is functioning correctly (Figure 3). Stimulations are then conducted after each operative phase: at the end of the incision, after positioning the implants, after positioning each rod and at the end of the corrective surgery. In cases of loss of signal (Figure 4), the patient’s vital signs and the anesthetic products dispensed are verified with the anesthetist and modified if necessary. If the anomalies persist, a Stagnara Wake up test is performed to assess the patient’s active clinical motricity [15].

### 2.3. Statistical Analysis

Demographic and radiographic measure was presented, such as mean, standard deviation (SD) and the range. The number of cases (and percentage) in which monitoring by the surgeon module could be achieved were reported and then analyzed according to the sites where the motor signal was collected. The sensitivity for detecting a potential neurological anomaly by the surgeon direct module MEP is expressed as the percentage value of the ratio of true positives (i.e., one case) to the total number of subjects who had clinically diagnosed neurological abnormalities due to the surgical procedures (i.e., one true positive and none false negative). The specificity is expressed as the percentage value of the ratio of true negatives (i.e., 178 cases) to the total number of subjects who had no clinically diagnosed neurological abnormalities due to the surgical procedures (i.e., 178 true negative + one false positive). The negative predictive value is expressed as the percentage of the ratio of true negatives (178 cases) to the total number of subjects whose neuromonitoring surgeon module did not detect any neurological abnormality (178 true negatives + none false negatives).

## 3. Results

### 3.1. Feasibility

The monitoring data was usable in 180 cases (94.7%). It was unusable in 10 cases, either because the response voltages were too low, there was no effective stimulation, there were aberrant responses or there was an initial response only in the upper limbs. This occurred in two idiopathic scoliosis cases that had uninterpretable responses because of an inappropriate anesthesia protocol. These two cases occurred at the beginning of our experience. The other case was an early idiopathic scoliosis with uninterpretable responses due to the immaturity of the nervous system and insufficient cortical stimulation. The other seven cases were secondary scoliosis (five cerebral palsy and two myopathies). Concerning the 180 usable cases, 99 patients (55%) had analyzable and reproducible responses at 8 receptor sites. 60 cases (33%) had responses at 6 of the 8 sites, 13 cases (7%) had responses at 4 of the 8 sites, and 8 cases (5%) had only 2 reproducible and symmetric sites on the lower limbs but with no analyzable response on the upper limbs. The analysis of the responses in function to the muscle group showed that usable responses were more frequent for the tibialis anterior muscle (93% of cases). The vastus lateralis muscle only provided usable responses in 56% of cases. In 85% of cases, we obtained usable responses in the control muscle group (thenar) and in the adductor hallucis group.

### 3.2. Safety and Reliability Study

The sensitivity for detecting a potential neurological anomaly was 100%, and the specificity was 99%. The predictive negative value was 100%. In the 180 cases where the monitoring data was usable, we found 178 true negatives, no false negatives and one false positive that required a wake-up test with recovery of evoked potentials occurring before the patient was actually awake. There was one true positive case with regressive partial tetraplegia in a patient who had undergone multiple operations. Three patients had a postoperative neurological examination that was not equivalent to their preoperative examination but not detectable by the monitoring (one loss of strength in the upper limbs in myopathy, one L4 radicular sensory deficit, one sensory deficit by compression on early mobilization of the interbody cage). These were not a result of a failure in the monitoring since such anomalies cannot be detected by the monitoring method used.

## 4. Discussion

To our knowledge, there are two recent publications reporting the results of “surgeon-directed module” NIM ECLIPSE for spinal surgery in children [11,12]. Magampa et al. [11] reviewed 299 cases operated for spinal deformity and using the MEP transcranial surgeon-module as a neurological monitoring tool. 93.3% (versus 94.7% in our paper) had acceptable tracings throughout the operation and woke up with normal clinical neurological function. Also in their study, the negative predictive value was 100%. Their secondary analysis showed that deterioration of motor potentials was significantly higher in congenital scoliosis than in AIS. Chan et al. [12] assessed transcranial MEP surgeon-module in 142 deformity correction surgeries. They also reported a negative predictive value of 100% with three cases (2.11%) showing complete visual loss of signals which led to reversal of the surgical procedure.

Our study supports the results of the two previous studies [11,12]. Surgeon-directed transcranial MEP was feasible in 94.7% of cases (180 out of 190) and provides new information regarding the response of muscle groups to motor stimulation of the cortex. This study shows that in 95% of cases at least four muscle sites have a reliable response among the eight sites tested. Focus on idiopathic scoliosis, surgeon monitoring was feasible in 146 out of 149 cases (i.e., 98% of cases). Of these three cases that could not be performed, two occurred at the start of our experiment with an unsuitable anesthetic protocol. Over the last few years, several risk factors involving modifications to the nerve afferents have been evidenced [16]. Many types of medication can influence the functioning of the motor pathways, notably curare, halogen gas and lidocaine, etc. Conversely, spinal anesthesia (morphine or sufentanil) has no influence on medullary function. The patient’s vital signs, notably low blood pressure, can change the electrophysiological responses. These merit attentive surveillance and highlight the crucial need for collaboration with the anesthesia team and the use of a predefined anesthesia protocol.

Concerning secondary scoliosis, surgeon monitoring was feasible in 34 out of 41 cases (i.e., 83% of cases). This difference in etiology was not reported in the two previous articles [11,12]. Recently, Shrader et al. [17] showed that 20.8% of children (among a cohort of 304 cases) with cerebral palsy could not be monitored by transcranial MEPs during spinal arthrodesis surgery. Latency times are often increased in these patients, and the surgeon must be vigilant about the value of the simulation voltage at the risk of triggering an increase in seizure activity [17].

Our study reported one case of false positives and one case of true positives. The steps to follow in cases of MEP disappearance are well defined. First, the patient’s vital signs should be checked, as should the drugs being dispensed, which can be modified if necessary. In cases of persistent anomalies, a wake-up test should be conducted. Generally, in the absence of neurological lesions, simply reducing the drugs and maintaining mean arterial pressure (MAP) > 70–80 mm Hg makes it possible to recover satisfactory curves without waiting for the patient to wake up completely. This is called the “anesthetic fade” by Ushirozako et al. [18]. We consider this to be an electrophysiological wake-up test with no actual clinical wake-up.

Previous studies assessed the efficacy of the different neuromonitoring methods (somatosensory evoked potential, motor evoked potential, neurogenic motor evoked potential, D waves and pedicular screw testing) and recommended the multimodal neuromonitoring because it was demonstrated that SSEP had 92% sensitivity and 98% specificity in detecting postoperative neurological complications [9,10,19,20,21]. This technique makes it possible to use the association of several simultaneous monitoring modalities (for example SSEP and MEP or SSEP and NMEP), in the presence of a neurophysiologist or electrophysiologist. SSEP change from baseline was defined as a 10% increase in latency and/or 50% decrease in amplitude. The MEP change was defined as a modification of the baseline, unilaterally or bilaterally. As our study confirms, using a “surgeon-directed” module is not the most exhaustive surveillance method but the technique makes it possible to eliminate the systematic clinical wake-up test, which provides non-negligible benefits.

Our study has limitations. It is a retrospective study, but no prospective study has been performed in the literature. In addition, the choice between transcranial MEP surgeon-module and multimodal monitoring performed by a specialist was at the discretion of the chief surgeon. Most of the idiopathic scoliosis were flexible and of low magnitude, with a reducibility index of 2.22 on average (Cincinnati index). In our study, we had no cases of vertebral osteotomy or major scoliosis above 90° who may require complex surgery: two-stage surgery (anterior approach followed by posterior arthrodesis or halo gravity traction preparation or temporary internal distraction). Any such cases were operated under multimodal monitoring performed by a neurologist specialized in electrophysiology. Previous studies [11,12] using the transcranial MEP surgeon module have shown that it can be used in cases of complex deformities with safety and efficiency. In opposition, some authors show that a combined anterior/posterior approach has been associated with a significant increase in the risk of neurological lesion (OR, 0.23; 95% CI, 0.08 to 0.81; *p* = 0.02) [22]. With improved surgical techniques, two-stage surgery (anterior and posterior) is more prone to complications [23]. Several studies have demonstrated the effectiveness of IOM in vertebral resection surgery or in severe deformities [24,25]. But 41% of severe scoliosis (major cobb angle of 80 or more) showed intraoperative changes in SSEP neuromonitoring [26]. Based on our experience, we believe that is preferable to have a neurophysiologist present in complex deformities cases. Firstly, to obtain more reliable multimodal recordings that are less subject to anesthetic variation or homeostasis. Secondly, this allows the surgeon to concentrate on the complex surgical procedure itself [27]. Moreover, our mid-term clinical and radiographic follow-up was poorly reported. No quality-of-life scores were collected, and only the radiographic measurement of the post-operative Cobb angle was reported in Table 1. All patients were seen post-operatively (covering the period from day one to three months post-operatively) in line with the aim of the study, and only the analysis of the clinical examination during this period was reported in the results section.

Lastly, this study is a single-center study. The results might not be representative of other surgical centers, potentially limiting the external validity and applicability of the findings. This potential bias is mitigated by the large number of subjects included in our article, and that our results are similar to those of the other two prior studies [11,12] using different correction techniques (previously discussed).

## 5. Conclusions

Simplified transcranial MEP monitoring known as “surgeon-directed module” is usable, safe and reliable in surgery for moderate scoliosis after a learning curve. It is possible to achieve a reliable response in 95% of cases with at least four of the eight muscle sites tested and with a negative predictive value of 100%. Particular attention should be paid to cases of cerebral palsy where it is difficult to achieve a motor response of transcranial MEP. In such cases who require at least partially functional motor pathways, it may be worthwhile to schedule a preoperative consultation to analyze motor responses and confirm the feasibility of monitoring. Some neuromonitoring devices used by surgeons alone allow joint motor and somesthetic-evoked potentials, but their feasibility and training have yet to be evaluated, particularly in cases of severe deformity.

## Figures and Tables

**Table 1:** Primary radiographic profile of cohort by diagnosis.

Etiology		ADC * Coronal Pre-Operative	Cincinnati Correction Index	ADC * Coronal Post Op
Idiopathic	Average	49	2.17	16
	SD **	15	1.69	10
	Min-max	12–95	0.4–11	0–72
Secondary	Average	60	3.3	29
	SD **	22	3.3	21
	Min-max	23–115	1–18	6–80

* ADC: Cobb angle, ** SD: Standard deviation.

## Data Availability

The data presented in this study are available on request from the corresponding author. The data are not publicly available due to ethical restrictions.
